# Confidence level in diagnosing and treating skin disorders among Palestinian physicians: a cross- sectional study

**DOI:** 10.1186/s12875-026-03431-1

**Published:** 2026-06-30

**Authors:** Hamzeh Yacoub, Lareen Othman, Nataly Jomaa, Yazan Alhabil, Zeena Othman, Mayar Abughosh, Leyan Qasrawi, Silvia Halteh, Francis Daibes, Ahmed Alhaj, Zaid Yacoub

**Affiliations:** 1 Faculty of Medicine, Arab American University of Palestine, West Bank, Palestine; 2 Faculty of Medicine, Al-Quds University, Jerusalem, Palestine; 3 Palestinian Dermatology Nexus, Ramallah, Palestine; 4 Faculty of Medicine and Health Sciences, Department of Medicine, An-Najah University, Nablus, Palestine; 5 SurgiCore-Jerusalem Surgical Interest Group of Jerusalem, Faculty of Medicine, Al-Quds University, Jerusalem, Palestine

**Keywords:** Dermatology, Self-efficacy, Diagnostic confidence, Medical education, Primary care, Physicians, Skin disorders

## Abstract

**Background:**

Skin disorders are among the most prevalent conditions encountered in primary care, yet dermatology remains underrepresented in medical curricula worldwide. Non-dermatologist physicians frequently manage these conditions with limited training and exposure, potentially compromising diagnostic accuracy and patient outcomes. This study aimed to assess the level of confidence among Palestinian physicians in diagnosing and managing skin disorders and to identify the training-related factors associated with higher diagnostic self-efficacy.

**Methods:**

A cross-sectional study was conducted among 246 licensed medical doctors practicing in the West Bank, Palestine. Participants were recruited through convenience sampling via professional networks and selected healthcare facilities, with the sample predominantly comprising younger and early-career physicians. Data were collected using a structured, validated self-administered questionnaire through mixed modes (electronic and paper-based). The instrument assessed sociodemographic and professional characteristics, dermatology exposure and training, and diagnostic self-efficacy using Likert-scale items. Diagnostic self-efficacy was dichotomized into below average versus average or above. Descriptive statistics, chi-square tests, and multivariable logistic regression were performed using SPSS version 22. Ethical approval was obtained from AlQuds University Institutional Review Board.

**Results:**

Of 246 participating physicians, 74.4% were aged 30 years or younger and 58.9% were male. The majority were general practitioners (54.1%) working in hospital settings (79.7%). Most participants (58.9%) reported no formal dermatology training, and 42.3% encountered fewer than 10 dermatological cases in their careers. Most physicians rated their diagnostic skills as average (62.6%), with a mean diagnostic self-efficacy score of 2.92 ± 0.78. No meaningful associations were observed between most sociodemographic characteristics and diagnostic self- efficacy. Training-related factors significantly associated with higher self-efficacy included: dermatology training during medical school (81.2% vs. 64.3%; *p* = 0.038), exposure to more than three training methods (91.6% vs. 73.3%; *p* = 0.006), and confidence gained after training (85.6% vs. 63.9%; *p* = 0.003). In multivariable logistic regression, confidence after training remained an independent predictor of higher self-efficacy (OR = 3.21, 95% CI 1.29–8.11; *p* = 0.011). Dermatology rotations and lectures were identified as the most effective and most recommended training modalities.

**Conclusions:**

Palestinian physicians demonstrate only moderate diagnostic self-efficacy in dermatology, largely attributable to limited training and clinical exposure. Dermatology training, particularly when it meaningfully boosts confidence, is the most important predictor of diagnostic self-efficacy, irrespective of sociodemographic background. Integrating structured, multimodal dermatology education into undergraduate and postgraduate curricula is essential to improving physician competence and ultimately enhancing patient care in primary care settings.

**Supplementary Information:**

The online version contains supplementary material available at https://doi.org/10.1186/s12875-026-03431-1.

## Introduction

Skin disorders represent a significant public health concern due to their high prevalence, chronic nature, and impact on individuals’ physical and psychosocial well-being. Common conditions such as acne, eczema, and psoriasis contribute substantially to the global burden of disease, often requiring long-term medical management and places considerable strain on healthcare systems. According to the World Health Organization (WHO), skin conditions can affect up to 1.8 billion people at any given time and location [1]. These disorders are particularly prevalent in low- and middle-income regions, such as the Middle East and North Africa (MENA). The Global Burden of Disease (GBD) study in 2019 reported over 16.6 million cases of dermatitis in the MENA region alone [2].

Despite their widespread impact, skin diseases often receive less attention than other medical conditions. Evidence shows that many healthcare providers lack sufficient training in dermatology, leading to gaps in the recognition and management of common skin disorders [3]. Furthermore, continuous changes in lifestyle and environmental factors in recent years have introduced additional challenges in diagnosing and treating skin conditions. Currently, skin disorders rank as the fourth most common non-fatal disease globally, affecting approximately 25% of the world’s population [4].

The involvement of non-dermatologist physicians in diagnosing and treating skin-related conditions has notably increased, particularly in primary care settings where disease evaluation is first conducted by general practitioners [5]. Although dermatologists provide specialized care, many patients initially seek help at non-specialty clinics. Consequently, primary care providers often demonstrate several deficiencies in managing these cases. Identifying gaps in clinicians’ dermatological knowledge is important, as these gaps can lead to inappropriate referrals, delayed access to care, and missed opportunities for timely and cost-effective management [6]. Incorporating dermatological knowledge into primary care improves patient care by enabling more accurate initial assessments and ensuring timely, appropriate referrals to other specialties. The dynamic relationship between dermatology and other medical fields is therefore essential for optimizing patient outcomes [7].

## Methods

### Study design and population

This cross-sectional study was conducted among licensed medical doctors practicing in the West Bank, Palestine, between January and April 2026. The target population comprised physicians across all levels of clinical experience and specialty backgrounds, including general practitioners, residents, and specialists. Eligible participants were medical doctors who were currently engaged in clinical practice in any healthcare setting, including hospitals, community-based clinics, and private practices. There were no restrictions based on specialty, gender, age, or years of experience. Medical students and non-physician healthcare workers were excluded. Participants were recruited through a convenience sampling approach using physicians’ emails, and direct distribution in selected healthcare facilities across the West Bank. A total of 246 physicians completed the questionnaire and were included in the final analysis.

Data were collected using a structured, self-administered questionnaire administered through a mixed-mode approach, including both electronic and paper-based formats. The online version of the questionnaire was distributed via Google Forms through email while printed paper questionnaires were distributed in selected healthcare facilities to enhance participation and improve coverage. Participation was voluntary and anonymous. For the online survey, electronic informed consent was obtained prior to accessing the questionnaire. For paper-based responses, written informed consent was obtained before completion.

The questionnaire was primarily adapted from a previously published survey assessing dermatological self-efficacy, clinical exposure, and training, with additional items informed by a study assessing dermatological diagnostic confidence [3, 5]. Following adaptation, the questionnaire underwent expert review by three board-certified dermatologists and two public health researchers to assess its face and content validity. The experts evaluated the clarity, relevance, comprehensiveness, and appropriateness of the questionnaire items for the target physician population. Their recommendations were incorporated before pilot testing and finalization of the instrument. A pilot study involving 20 physicians was conducted to evaluate the clarity, relevance, and feasibility of the questionnaire, and minor modifications were made accordingly. As the instrument was intended primarily for descriptive assessment rather than as a multidimensional psychometric scale, formal factor analysis was not performed. The full questionnaire is provided in Appendix 1. It comprised three main sections:

1. Sociodemographic and Professional Characteristics:
  Including age, gender, marital status, years of clinical experience, medical title, work setting, department, and location of medical education.
2. Confidence and Self-Efficacy Assessment:
  Participants rated their confidence in diagnosing and teaching dermatological conditions using two five-point Likert-type items. The response options were coded as follows: 1 = much below average, 2 = below average, 3 = average, 4 = above average, and 5 = much above average. Higher scores therefore represented greater self-reported diagnostic or teaching ability.
  For the categorical association analyses, diagnostic self-efficacy responses were grouped into two categories: below average (scores 1-2) and average or above (scores 3-5). This grouping was used to identify factors associated with attaining at least an average level of perceived diagnostic ability and to provide a clear and practically interpretable comparison between participants who reported below average ability and those who reported average or higher ability.
3. Dermatology Exposure and Training:
  Assessing the number of dermatological cases encountered, exposure to specific conditions (e.g., atopic dermatitis), duration of dermatology training, and types of educational methods received (e.g., lectures, clinical rotations, case-based discussions). Training duration was measured using four ordered categories: none, less than one month, one to three months, and more than three months. This variable was treated as an ordinal categorical variable and was not considered a five-point Likert-type item.

### Data analysis

Data analysis was conducted using the Statistical Package for Social Sciences (SPSS) version 22. Answering all items was required to complete the questionnaires. Therefore, no missing data were present. Descriptive statistics, including frequencies and percentages for categorical variables, were calculated to summarize the data. Advanced analyses, such as Chi-square test of independence (χ² test), Fisher’s exact test, multiple linear regression and Pearson correlation, were performed to examine the relationships between variables. Statistical significance was set at *p* < 0.05, ensuring reliable results. This approach provided a clear understanding of the data and supported meaningful conclusions. Lastly, an internal consistency of the adapted instrument was assessed using Cronbach’s alpha in the study sample.

### Ethical consideration

Ethical approval for the study was obtained from AlQuds University Institutional Review Board (IRB) prior to data collection. Participation was voluntary, and informed consent was obtained electronically. All data were collected anonymously, and confidentiality was strictly maintained throughout the study.

## Results

### Participants’ characteristics

A total of 246 medical doctors participated in the study. The sociodemographic and professional characteristics of the participants are summarized in Table 1.

**Table 1:** Sociodemographic and professional characteristics of participants

Variable	Category	*n*	%
Age (years)	≤ 30	183	74.4
	30–40	40	16.3
	40–50	13	5.3
	50–60	8	3.3
	≥ 60	2	0.8
Gender	Male	145	58.9
	Female	101	41.1
Marital status	Single	156	63.4
	Married	69	28.0
	Divorced	3	1.2
	Other / Prefer not to say	18	7.3
Years in clinical practice	< 1 year	109	44.3
	1–5 years	85	34.6
	6–10 years	21	8.5
	11–15 years	8	3.3
	> 15 years	23	9.3
Medical title	General practitioner	133	54.1
	Resident	63	25.6
	Specialist	50	20.3
Current work setting*	Hospital	196	79.7
	Private practice	63	25.6
	Community-based clinic	37	15.0
Departmental setting	Medical	93	37.8
	Surgical	82	33.3
	Primary healthcare	71	28.9
Medical school location	Palestine	155	63.0
	Abroad	91	37.0

*Multiple responses possible

Most participants were ≤ 30 years of age (74.4%) and male (58.9%).

Regarding professional background, most respondents had limited clinical experience, with 44.3% reporting less than one year of practice and 34.6% reporting 1–5 years of experience. Over half of the participants were general practitioners (54.1%), followed by residents (25.6%) and specialists (20.3%). Most respondents worked in hospital settings (79.7%), while smaller proportions worked in private practice (25.6%) or community-based clinics (15.0%). In terms of departmental distribution, participants were relatively evenly distributed between medical departments (37.8%), surgical departments (33.3%), and primary healthcare settings (28.9%). Most participants had graduated from medical schools in Palestine (63.0%), while 37.0% had obtained their medical degrees abroad. 

Table 2 summarises the participants’ clinical exposure to dermatological conditions, training duration, and self-reported confidence in diagnosing and teaching dermatological diseases.

**Table 2:** Clinical exposure, training duration, and self-efficacy in diagnosing and teaching dermatological conditions

Variable	Category	*n*	%
Encounter with any dermatological case	< 10	104	42.3
	10–20	67	27.2
	20–40	41	16.7
	> 40	34	13.8
Encounter with atopic dermatitis cases	< 10	162	65.9
	10–20	51	20.7
	20–40	20	8.1
	> 40	13	5.3
Training Duration	None	145	58.9
	< 1 month	69	28.0
	1–3 months	28	11.4
	> 3 months	4	1.6
Diagnosis skills	Much below average	14	5.7
	Below average	37	15.0
	Average	154	62.6
	Above average	36	14.6
	Much above average	5	2.0
Teaching skills	Much below average	16	6.5
	Below average	78	31.7
	Average	122	49.6
	Above average	27	11.0
	Much above average	3	1.2

Mean diagnostic score: 2.92 ± 0.78

Mean teaching score: 2.69 ± 0.80

In terms of exposure to dermatological cases overall, 42.3% (*n* = 104) of participants said they had seen fewer than 10 cases, and 27.2% (*n* = 67) said they had seen 10–20 cases. Smaller proportions reported seeing 20–40 cases (16.7%, *n* = 41) or more than 40 cases (13.8%, *n* = 34).

With respect to exposure specifically to atopic dermatitis cases, the majority of participants (65.9%, *n* = 162) reported encountering fewer than 10 cases. In comparison, 20.7% (*n* = 51) had encountered 10–20 cases, 8.1% (*n* = 20) reported seeing 20–40 cases, and only 5.3% (*n* = 13) reported encountering more than 40 cases.

In terms of dermatology training duration, most respondents reported having received no formal training (58.9%, *n* = 145). Meanwhile, 28.0% (*n* = 69) had received less than one month of training, 11.4% (*n* = 28) reported training lasting 1–3 months, and only 1.6% (*n* = 4) trained for exceeding three months. The participants evaluated their dermatological diagnostic skills, with most (62.6%, *n* = 154) classifying their abilities as average. Conversely, 14.6% (*n* = 36) stated above-average proficiency, whereas 2.0% (*n* = 5) stated much above-average proficiency. In contrast, 15.0% (*n* = 37) assessed their skills as below average, while 5.7% (*n* = 14) classified them as much below average. Similarly, participants were asked to assess their teaching abilities in dermatological subjects. Approximately 49.6% of the respondents classified their teaching abilities as average (*n* = 122); 31.7% (*n* = 78) indicated below-average skills, while 6.5% (*n* = 16) classified their skills as much below average. However, minor proportions were classified as having above-average (11.0%, *n* = 27) or much above-average teaching abilities (1.2%, *n* = 3).

The means and standard deviations are provided as supplementary descriptive summaries of the five-point ratings. Frequencies and percentages constitute the primary summaries of these ordinal responses.

**Fig. 1:**
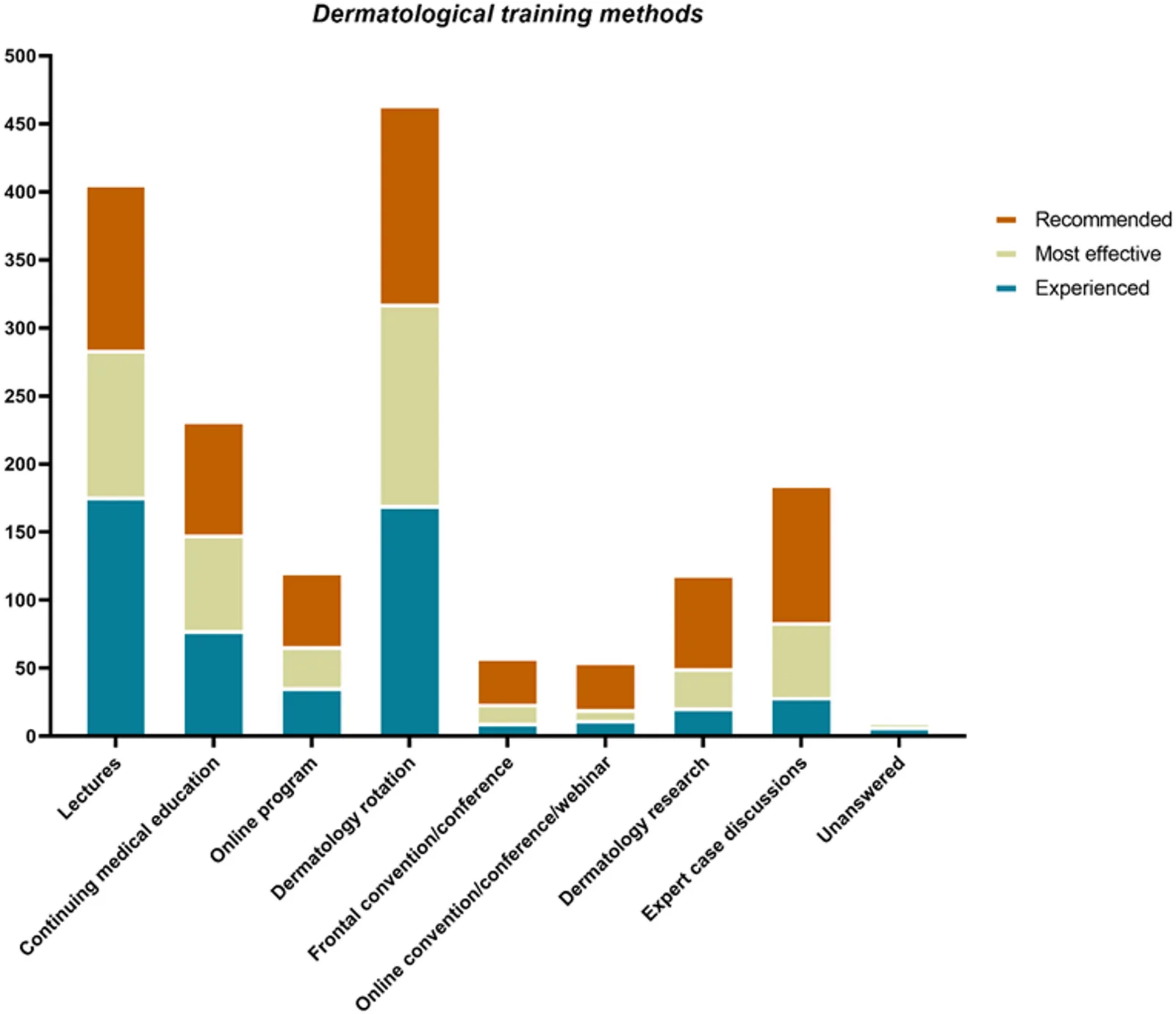
Experienced, most effective and recommended dermatological training methods according to participants

Figure 1 shows the participants’ experiences, their evaluations of methods’ effectiveness, and their recommendations for dermatology training methods.

Lectures (71.5%) and dermatology rotations (69.1%) were the most common training methods used. Furthermore, dermatology rotations were perceived as the most effective approach (60.1%), followed by lectures (43.9%). Regarding recommendations, dermatology rotations (59.3%) and lectures (49.6%) were again the most frequently endorsed methods. Although expert case discussions were less commonly experienced (11.8%), they were deemed effective by 22.4% of participants and were recommended by 41.1%. Continuing medical education demonstrated moderate engagement across all assessed categories. Despite encountering online programs and dermatology research less frequently, a significant percentage of participants still endorsed these methods (22.4% and 28%, respectively). On the other hand, frontal conferences and online webinars were the least experienced, least effective and least recommended training methods. Overall, participants perceived clinical dermatology rotations and traditional lectures as the most effective methods for dermatology training.

Associations of participants’ characteristics with diagnostic self-efficacy.

Tables 3 and 4 show the associations between participants’ sociodemographic characteristics, professional background, dermatology exposure, and training factors with self-efficacy in diagnosing dermatological conditions.

**Table 3:** Association between sociodemographic and professional characteristics and self-efficacy in diagnosing dermatological conditions among participating physicians

Variable	Category	Self-Efficacy < Average *n* (%)	Self-Efficacy ≥ Average *n* (%)	*p*-value
Age (years)	≤ 30	39 (21.3)	144 (78.7)	0.739
	30–40	7 (17.5)	33 (82.5)	
	40–50	3 (23.1)	10 (76.9)	
	50–60	2 (25.0)	6 (75.0)	
	≥ 60	0 (0.0)	2 (100)	
Gender	Female	21 (20.8)	80 (79.2)	0.984
	Male	30 (20.7)	115 (79.3)	
Marital status	Single	32 (20.5)	124 (79.5)	0.028**
	Divorced	2 (66.7)	1 (33.3)	
	Married	10 (14.5)	59 (85.5)	
	Other / Prefer not to answer	7 (38.9)	11 (61.1)	
Years in clinical medicine	< 1 year	25 (22.9)	84 (77.1)	0.328
	1–5 years	18 (21.2)	67 (78.8)	
	6–10 years	3 (14.3)	18 (85.7)	
	11–15 years	1 (12.5)	7 (87.5)	
	> 15 years	4 (17.4)	19 (82.6)	
Medical title	General practitioner	27 (20.3)	106 (79.7)	0.968
	Resident	13 (20.6)	50 (79.4)	
	Specialist	11 (22.0)	39 (78.0)	
Current work setting	Hospital	39 (19.9)	157 (80.1)	0.797
	Community-based clinic	8 (21.6)	29 (78.4)	
	Private practice	31 (49.2)	32 (50.8)	
Current department	Medical	24 (25.8)	69 (74.2)	0.186
	Primary healthcare	10 (14.1)	61 (85.9)	
	Surgical	17 (20.7)	65 (79.3)	
Medical school location	Abroad	23 (25.3)	68 (74.7)	0.178
	Palestine	28 (18.1)	127 (81.9)	

** *P*-value ≤ 0.05

**Table 4:** Association between dermatology-related clinical exposure and training factors and self-efficacy in diagnosing dermatological conditions among participating physicians

Variable	Category	Self-Efficacy < Average *n* (%)	Self-Efficacy ≥ Average *n* (%)	*p*-value
Facility has dermatology department	Yes	25 (20.2)	99 (79.8)	0.824
	No	26 (21.3)	96 (78.7)	
Encounter with dermatological cases	< 10	11 (16.4)	56 (83.6)	0.052
	10–20	7 (17.1)	34 (82.9)	
	20–40	4 (11.8)	30 (88.2)	
	> 40	29 (27.9)	75 (72.1)	
Encounter with atopic dermatitis cases	< 10	8 (15.7)	43 (84.3)	0.388
	10–20	3 (15.0)	17 (85.0)	
	20–40	4 (30.8)	9 (69.2)	
	> 40	36 (22.2)	126 (77.8)	
Dermatology training in medical school	No	10 (35.7)	18 (64.3)	0.038**
	Yes	41 (18.8)	177 (81.2)	
Dermatology training in residency	No	20 (22.2)	70 (77.8)	0.392
	Yes	13 (26.0)	37 (74.0)	
Number of dermatology training methods	1–2	43 (26.7)	118 (73.3)	0.006**
	> 3	7 (8.3)	77 (91.6)	
Confidence after training	Yes	25 (14.4)	149 (85.6)	0.003**
	No	26 (36.1)	46 (63.9)	

** *P*-value ≤ 0.05

No consistent associations were observed between sociodemographic characteristics and diagnostic self-efficacy, suggesting that educational exposure and training experiences may play a more important role than demographic factors in shaping physicians’ confidence in diagnosing dermatological conditions.

Regarding dermatology-related exposure and training factors, several variables showed significant associations. In addition, dermatology training during medical school was associated with higher self-efficacy, as participants who received training were more likely to report above or equal average self-efficacy compared with those who did not (81.2% vs. 64.3%, *p* = 0.038). Similarly, participants who reported receiving more than three dermatology training methods had higher self-efficacy levels than those who received one or two methods (91.6% vs. 73.3%, *p* = 0.006). Finally, confidence after training was also significantly associated with higher self-efficacy (85.6% vs. 63.9%, *p* = 0.003).

In multivariable logistic regression analysis, higher confidence after dermatology training remained independently associated with higher diagnostic self-efficacy (OR = 3.21, 95% CI 1.29–8.11, *p* = 0.011). In the stratified analysis, receiving more than three dermatology training methods (OR = 6.55, 95% CI 1.15–7.41, *p* = 0.008), consulting dermatologists only for unclear cases (OR = 4.39, 95% CI 1.10–5.86, *p* = 0.033), and referring to medical literature (OR = 3.99, 95% CI 1.16–4.92, *p* = 0.039) were also associated with higher self-efficacy.

Lastly, the adapted instrument demonstrated good internal consistency in the present sample of general physicians. The overall Cronbach’s alpha was 0.83. Subscale reliability coefficients ranged from 0.77 to 0.86, indicating acceptable to good reliability across domains.

## Discussion

### Summary of key findings

This study evaluated the association between dermatology training and diagnostic self-efficacy among Palestinian physicians. Most physicians reported limited exposure to and training in dermatology and felt their diagnostic skills were average overall. Dermatology lectures and rotations were viewed as the most effective educational modalities, and confidence gained after training was identified as the strongest predictor for increased diagnostic self-efficacy. The results indicate that formal training in dermatology has value in increasing physician confidence in diagnosing skin conditions.

### Dermatology exposure and training

According to this study, a sizable portion of physicians had limited experience with dermatological conditions and no formal training in dermatology. Specifically, more than half (58.9%) of the participants reported having no formal training in dermatology, and the majority had seen fewer than 20 dermatological cases in their clinical practice. This limited exposure is likely the cause of the participants’ moderate levels of diagnostic self-efficacy. As a visually orientated speciality, dermatology primarily relies on pattern recognition, which is learned through repeated clinical experience as opposed to merely theoretical instruction [8]. Therefore, insufficient exposure during training may hinder the development of diagnostic competence [9].

These findings are consistent with other studies that demonstrate that, despite dermatological disorders accounting for a significant portion of primary care consultations, dermatology is still under-represented in many undergraduate medical curricula [10].Poor clinical exposure and a lack of curriculum time are global issues, according to research, which may lead to deficiencies in physicians’ ability to recognize and treat common skin conditions [11].

### Self-efficacy in diagnosing dermatological conditions”

The majority of doctors in this survey rated their diagnostic skills as “average” (62.6%), with relatively few reporting above-average or high proficiency. This pattern suggests that physicians may only be moderately confident in their ability to diagnose dermatological conditions.

The fact that dermatology is a speciality that requires clinical knowledge and strong visual diagnostic skills can help to explain this outcome. Unlike other specialities that may rely more on laboratory or imaging results, dermatology primarily depends on the clinical identification of lesions. Therefore, a lack of exposure and training may have a direct effect on how competent doctors are perceived [12].

Previous studies have demonstrated that structured training programs and increased exposure to clinical cases significantly enhance diagnostic accuracy in dermatology [13].Therefore, the moderate level of self-efficacy observed in this study may reflect gaps in both undergraduate and postgraduate dermatology education.

### Effectiveness of dermatology training methods

This study revealed that the most widely used and regarded as the most effective training methods in dermatology were lectures and rotations. For instance, dermatology rotations were deemed the most effective strategy by 60.1% of participants.

Interestingly, participants highly valued and recommended expert case talks, despite their rarity. This suggests that there is a difference between the teaching methods that are currently employed and those that are believed to be the most beneficial.

Clinical rotations are advantageous because they allow students to examine and diagnose real-world situations, which improves their capacity to identify patterns and apply clinical reasoning. Similarly, lectures provide basic knowledge that supports clinical understanding [14].

These findings are in line with earlier studies that emphasise the importance of experiential and case-based learning in dermatological education [15]. Case discussions and interactive learning techniques have been shown to enhance diagnostic accuracy and knowledge retention, particularly in visually stimulating specialities like dermatology [16].

### Factors associated with higher diagnostic self-efficacy

A number of training parameters related to dermatology showed a significant correlation with higher diagnostic self-efficacy. These included receiving dermatology training during medical school, being exposed to a variety of training methods, and gaining confidence after training.

Interestingly, multivariable regression analysis revealed that confidence after dermatology training remained an independent predictor of higher diagnostic self-efficacy (OR = 3.21, *p* = 0.011). This finding highlights the significance of perceived ability in influencing physicians’ confidence in their diagnosis.

The association between multiple training modalities and higher self-efficacy suggests that diverse educational approaches may enhance learning outcomes. Combining lectures, clinical rotations, and case-based discussions probably improves retention and strengthens knowledge.

Additionally, the high predictive value of confidence after training highlights how important it is to ensure that training programs effectively boosts learners’ confidence in applying that knowledge in clinical settings [17, 18].

These findings support educational theories that link self-efficacy to performance, suggesting that physicians who are more self-assured are more likely to successfully engage in clinical decision-making and diagnostic processes [19].

### Unexpected and notable findings

Most sociodemographic characteristics such as age, gender, professional title, workplace setting, geographic location, and years of clinical experience did not appear to influence clinicians’ confidence in diagnosing dermatological conditions.

Similar findings have been reported in previous studies, suggesting that dermatology-related training and educational exposure may play a more important role in shaping physicians’ confidence than demographic characteristics [20].

### Implications

The results of this study have important implications for medical education and clinical practice. They highlight limited exposure to dermatology and insufficient structured training among physicians. Given the high prevalence of skin conditions in primary care, this gap may negatively impact diagnostic accuracy and patient management [21].

Consistent with previous literature, structured dermatology training is associated with improved diagnostic confidence and competence [10].This underscores the importance of integrating formal dermatology education into medical curricula, with early and continuous exposure to clinical cases to support skill development.

The findings further suggest that teaching methods significantly influence physician confidence. A combination of lectures, interactive learning, and hands-on clinical experience appears more effective than traditional didactic approaches alone. Clinical exposure supports the development of pattern recognition skills essential for dermatological diagnosis, while case-based discussions enhance clinical reasoning and should be more widely incorporated into training programs [15, 16].

Importantly, the study indicates that confidence gained through training is closely linked to perceived diagnostic ability [19]. Therefore, medical education should emphasize not only knowledge acquisition but also the development of clinical confidence through supervised practice, feedback, and active learning strategies [22].

Overall, strengthening dermatology training for non-specialist physicians may improve early recognition and management of skin conditions, reduce unnecessary referrals, and enhance efficiency in primary care settings where such conditions are commonly encountered [23].

### Strengths and limitations

This study has several strengths. To the best of our knowledge, it is among the first to systematically assess dermatological diagnostic self-efficacy among physicians in Palestine, thereby addressing an important gap in the regional literature. The use of a mixed-mode data collection strategy, combining electronic and paper-based questionnaires, likely enhanced participation and improved representation across diverse healthcare settings. In addition, the questionnaire was adapted from previously validated instruments and underwent expert review and pilot testing, supporting its content validity. The inclusion of physicians from multiple professional levels and practice environments, including general practitioners, residents, specialists, hospital-based, community, and private sector clinicians, enhances the breadth and clinical relevance of the findings.

Several limitations should also be considered when interpreting the results. The cross-sectional design precludes causal inference, and observed associations between training-related variables and self-efficacy cannot be interpreted in terms of directionality. The study relied on self-reported measures of diagnostic confidence, which may not accurately reflect actual diagnostic performance in clinical practice. Objective assessment methods such as clinical vignettes, image-based testing, or standardized diagnostic evaluations were not included, limiting the ability to correlate self-efficacy with true diagnostic accuracy. In addition, the use of a non-probability convenience sampling strategy, partly through professional networks and selected healthcare facilities, introduces the possibility of selection bias. Physicians with a particular interest in dermatology or medical education may have been more likely to participate, which may limit representativeness.

Furthermore, the sample was disproportionately composed of young and early-career physicians, with a large proportion aged 30 years or younger and reporting limited clinical experience. This distribution likely reflects the sampling approach and may have led to an overrepresentation of junior clinicians. As a result, the findings may be more reflective of dermatology-related training experiences and confidence levels among early-career physicians than among more experienced practitioners. Finally, social desirability bias cannot be excluded, as participants may have provided responses perceived as more favorable despite assurances of anonymity.

In addition, although the questionnaire was adapted from previously validated instruments and underwent expert review and pilot testing, formal psychometric validation of the modified version, including exploratory or confirmatory factor analysis, was not conducted. Therefore, the extent to which the original factor structure was preserved following adaptation cannot be fully determined.

### Future research directions

While this study provides valuable insights into dermatology training and diagnostic self-efficacy among physicians, it also highlights several important directions for future research. Subsequent studies should address methodological limitations and focus on evaluating effective educational strategies.

### Moving from self-reported confidence to objective assessment

This study identified a substantial lack of formal dermatology training (58.9%) and limited clinical exposure, with most physicians reporting only moderate diagnostic confidence (62.6%). These findings align with previous research showing similar gaps in dermatology education among non-specialist physicians, likely reflecting the underrepresentation of dermatology in undergraduate curricula [21, 22].

However, as this study relies on self-reported confidence, future research should focus on objective assessment of diagnostic ability. Standardized clinical examinations, image-based assessments, or OSCEs are needed to determine whether perceived confidence accurately reflects diagnostic accuracy in clinical practice.

The current study population consisted mainly of young general practitioners trained in Palestine and working in hospital settings. While the findings are consistent with global evidence on dermatology training gaps, physician exposure and competence may vary depending on healthcare structure, curriculum design, and referral systems.

Future research should therefore expand across different countries and healthcare settings to improve generalizability and identify best practices for integrating dermatology into medical education. Comparative studies across regions would be particularly useful in determining how system-level differences influence training quality and diagnostic competence.

### Evaluating structured educational interventions

Given the widespread training gaps identified, future research should shift toward evaluating educational solutions. This study found that exposure to multiple training methods was associated with higher diagnostic confidence (OR = 6.55). In addition, although expert case discussions were rarely experienced, they were frequently recommended by participants, suggesting a perceived educational need.

Future studies should therefore assess the effectiveness of structured dermatology training programs, particularly multimodal and case-based learning approaches. Further research should also consider how outcomes vary according to learners’ baseline clinical experience, as a considerable proportion of participants were early-career physicians.

### Longitudinal evaluation of training impact

The finding that sustained confidence after training is associated with higher diagnostic self-efficacy (OR = 3.21) highlights the importance of long-term evaluation. However, cross-sectional designs cannot determine whether these effects persist over time.

Future research should adopt longitudinal designs to assess the durability of dermatology training effects. Following cohorts from medical school through residency and clinical practice would help determine whether diagnostic skills are maintained, improved, or decline over time, and would inform the optimal timing of continuing medical education.

## Conclusion

This study demonstrates that physicians in Palestine have limited exposure to dermatology and, consequently, only moderate levels of diagnostic self-efficacy in managing skin disorders. Most participants reported no formal dermatology training and had encountered fewer than 20 dermatological cases in clinical practice, which likely contributes to the moderate confidence levels observed. These findings are consistent with global evidence highlighting the underrepresentation of dermatology in undergraduate and postgraduate medical curricula.

Dermatology training emerged as the most significant determinant of diagnostic self-efficacy. Physicians who received dermatology training during medical school, were exposed to multiple training modalities, and reported an increase in confidence following training were significantly more likely to report higher self-efficacy. Notably, confidence gained through training was an independent predictor of diagnostic self-efficacy, emphasising that effective training must translate into genuine clinical confidence. Sociodemographic factors, including age, gender, years of experience, and medical title, were not associated with self-efficacy, underscoring that training quality outweighs individual background characteristics.

These findings have direct implications for medical education policy. Integrating structured, clinically focused dermatology training combining lectures, supervised clinical rotations, and case-based discussions into both undergraduate and postgraduate curricula is essential for improving physician competence in managing skin disorders. Strengthening dermatology education for non-specialist physicians has the potential to improve early recognition of skin conditions, reduce diagnostic delays, limit unnecessary referrals, and ultimately enhance patient outcomes in primary care settings where dermatological conditions are frequently encountered.

## Supplementary Information

Supplementary Material 1.

## Data Availability

The dataset used and/or analysed during the current study are available from the corresponding author on reasonable request.
