# Supplementary material for: Confidence level in diagnosing and treating skin disorders among Palestinian physicians: a cross- sectional study
Source: BMC Prim Care. 2026 Jun 30;27:336. doi: 10.1186/s12875-026-03431-1 (PMC13591905; doi:10.1186/s12875-026-03431-1)
Supplement: Supplementary file 1 [file 12875_2026_3431_MOESM1_ESM.docx]

**Confidence Level in Diagnosing and Treating Skin Disorders**

**among Palestinian Physicians**

**Information for Participants & Informed Consent**

You are invited to participate in a research study that aims to assess the confidence level of physicians in Palestine in diagnosing and treating common skin disorders. Before deciding whether to participate, it is important to understand the purpose of the study, what your participation involves, and your rights as a participant.

The goal of this study is to evaluate how confident Palestinian physicians feel when diagnosing and managing various dermatological conditions. The findings will help identify areas where additional training or resources may be needed to improve the quality of dermatological care.

• You will be asked to complete a questionnaire.

• The questionnaire includes questions about your demographic background, clinical experience, and your confidence in dealing with specific skin disorders.

• The survey will take approximately 5 - 10 minutes to complete.

• Participation is totally voluntary and anonymous.

• All responses will be anonymous. No identifiable personal information will be collected. Data will be stored securely and used only for academic and scientific purposes. Results may be published in scientific journals or presented at conferences, but no individual participant will be identifiable.

**Please note that your participation is completely voluntary, and you may choose not to answer any question or to stop participating at any time.**

*Research Team (supervised by Dr. Hamzeh Yacoub.* [*hamzehkifah8@gmail.com*](mailto:hamzehkifah8@gmail.com)*, +970598959064)*

**Consent**

**By signing below and continuing to the survey, you confirm that:**

1. You voluntarily agree to participate in this study.

2. You are a physician currently practicing in Palestine. *****

**☐ I agree**

Participant Signature: ________________________________________ Date: ____________________

**Section 1: Demographic Questions**

*Please complete the following questions according to your current status:*

**3. Age ***

*Mark only one oval.*

○	30 or under

○	30 – 40

○	40 – 50

○	50 – 60

○	60 and above

**4. Gender ***

*Mark only one oval.*

○	Male

○	Female

**5. Religion ***

*Mark only one oval.*

○	Christian

○	Muslim

○	Other / Do not wish to answer

**6. Marital Status ***

*Mark only one oval.*

○	Single

○	Married

○	Divorced

○	Widower

○	Other / Do not wish to answer

**7. Medical Status ***

*Mark only one oval.*

○	Resident

○	Specialist

○	General Practitioner (GP)

**8. If resident, then: which year of residency?**

*Mark only one oval.*

| **1** | **2** | **3** | **4** | **5** | **6** |
| --- | --- | --- | --- | --- | --- |
| ○ | ○ | ○ | ○ | ○ | ○ |

**9. How many years of clinical experience do you have as a physician? ***

*Mark only one oval.*

○	Less than 1 year

○	1–5 years

○	6–10 years

○	11–15 years

○	More than 15 years

**10. Does your medical facility (e.g. hospital, clinic…) have a dermatology department? ***

*Mark only one oval.*

○	Yes

○	No

**11. Current place of work (you can choose more than one) ***

*Check all that apply.*

☐	Hospital

☐	Community based clinic

☐	Private practice

**12. Working department ***

*Mark only one oval.*

○	Primary health care

○	Internal Medicine

○	Pediatrics

○	Surgery

○	OBGYN

○	Other: _____________________________________________

**13. Where did you attend medical school? ***

*Mark only one oval.*

○	Palestine

○	Europe

○	USA / Canada

○	Egypt

○	Jordan

○	Syria

○	Turkey

○	Iraq

○	Eastern Europe

○	Western Europe

○	Australia

○	Arab Gulf

○	Sudan

○	Other: _____________________________________________

**14. Place of Medical Residency (for residents and specialists)**

*Mark only one oval.*

○	Palestine

○	Egypt

○	Jordan

○	Syria

○	Turkey

○	Iraq

○	Eastern Europe

○	Western Europe

○	United States / Canada

○	Australia

○	Arab Gulf

○	Africa

**15. In your estimation, how many patients with a skin disorder have you encountered in the last year? ***

*Mark only one oval.*

○	Under 10

○	10–20

○	20–40

○	Above 40

**16. In your estimation, how many patients with Atopic Dermatitis have you encountered in the last year? ***

*Mark only one oval.*

○	Under 10

○	10–20

○	20–40

○	Above 40

**Section 2: Self-Efficacy Questions**

The following questions address the self-assessment of your skills with managing and treating skin disorders, particularly atopic dermatitis in the general patient population.

Please rate the following 3 questions on a scale of 1–5 (1 represents much below average, and 5 represents much above average).

**17. Please rate the following on a scale of 1–5: ***

*Mark only one oval per row.*

|  | **Much below average** | **Below average** | **Average** | **Above average** | **Much above average** |
| --- | --- | --- | --- | --- | --- |
| How do you estimate your skills in diagnosing and managing a skin disorder in your patient population? | ○ | ○ | ○ | ○ | ○ |
| How do you estimate your ability to teach colleagues to manage skin disorders in your patient population? | ○ | ○ | ○ | ○ | ○ |

**18. Please rate the following statements on a scale of 1–5 (1 = Strongly disagree, 5 = Strongly agree): ***

*Mark only one oval per row.*

|  | **Strongly disagree** | **Disagree** | **Neutral** | **Agree** | **Strongly agree** |
| --- | --- | --- | --- | --- | --- |
| I would use online search engine to manage a case of a skin disorder | ○ | ○ | ○ | ○ | ○ |
| I would use medical literature information to manage a case of a skin disorder | ○ | ○ | ○ | ○ | ○ |
| I would consult/refer to a dermatologist every case of a skin disorder | ○ | ○ | ○ | ○ | ○ |
| I would consult/refer to a dermatologist only in a case of unclear diagnosis | ○ | ○ | ○ | ○ | ○ |
| I would consult/refer to a dermatologist skin cases because of parents' anxiety | ○ | ○ | ○ | ○ | ○ |

**Section 3: Education Tool**

The following questions refer to dermatology education methods. Please answer yes or no in the appropriate questions and choose from the multiple-choice questions according to your experience.

**19. Have you had dermatology training as part of your medical school or internship? ***

*Mark only one oval.*

○	Yes

○	No

**20. Have you had dermatology training during your residency? (for residents and specialists only) ***

*Mark only one oval.*

○	Yes

○	No

**21. If yes, what is/was the duration?**

*Mark only one oval.*

○	Less than 1 month

○	1–3 months

○	More than 3 months

**22. What is the method of training you have experienced? You may choose more than one answer. ***

*Check all that apply.*

☐	Lectures

☐	Continuing medical education

☐	Online program

☐	Dermatology rotation

☐	Frontal convention / Conference

☐	Online convention / Conference / Webinar

☐	Dermatology research

☐	Experts case discussions

☐	Other: _____________________________________________

**23. Have you felt more confident in treating skin disorders in your patient population after experiencing those programs? ***

*Mark only one oval.*

○	Yes

○	No

○	Other: _____________________________________________

**24. Which method was most effective in improving your dermatologic abilities? You may choose more than one answer. ***

*Check all that apply.*

☐	Lectures

☐	Continuing medical education

☐	Online program

☐	Dermatology rotation

☐	Frontal convention / Conference

☐	Online convention / Conference / Webinar

☐	Dermatology research

☐	Experts case discussions

☐	Other: _____________________________________________

**25. From the methods listed below, which would you recommend adding as a mandatory curriculum in a residents training program that would positively affect their dermatology skills? You may choose more than one answer. ***

*Check all that apply.*

☐	Lectures

☐	Continuing medical education

☐	Online program

☐	Dermatology rotation

☐	Frontal convention / Conference

☐	Online convention / Conference / Webinar

☐	Dermatology research

☐	Experts case discussions

☐	Other: _____________________________________________

**Thank you for your participation.**

*All responses are completely anonymous and confidential.*
